# Recent advances in hypervalent iodine(III)-catalyzed functionalization of alkenes

**DOI:** 10.3762/bjoc.14.154

**Published:** 2018-07-18

**Authors:** Xiang Li, Pinhong Chen, Guosheng Liu

**Affiliations:** 1 State Key Laboratory of Organometallic Chemistry, Center for Excellence in Molecular Synthesis, Shanghai Institute of Organic Chemistry, Chinese Academy of Sciences, 345 Lingling Road, Shanghai 200032, China

**Keywords:** asymmetric catalysis, functionalization of alkenes, hypervalent iodine(III)

## Abstract

Hypervalent iodine(III) reagents have been well-developed and widely utilized in functionalization of alkenes, however, generally either stoichiometric amounts of iodine(III) reagents are required or stoichiometric oxidants such as *m*CPBA are employed to in situ generate iodine(III) species. In this review, recent developments of hypervalent iodine(III)-catalyzed functionalization of alkenes and asymmetric reactions using a chiral iodoarene are summarized.

## Introduction

**Figure 1:**
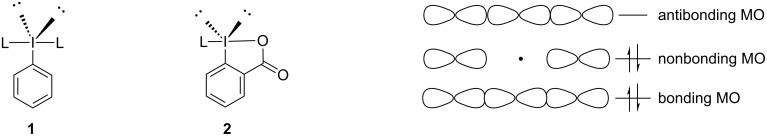
The structures of hypervalent iodine (III) reagents [8].

Hypervalent iodine(III) reagents, also named as λ^3^-iodanes, have been widely used in organic synthesis since the 1990s, due to their stability, low toxicity and mild reaction conditions [1–10]. Structurally, they always adapt a distorted trigonal bipyramidal geometry in which the less electronegative aryl ring and two lone pairs of electrons are occupying the equatorial positions, and the electronegative ligands are in the apical positions (Figure 1, **1** and **2**) [8]. Hypervalent iodine(III) reagents are electrophile in nature, resulting from the node in a hypervalent nonbonding orbital, a 3-center-4-electron (3*c*-4*e*) bond (L–I–L), which is formed by the overlap of the 5p orbital of iodine atom with the orbitals of two ligands (Figure 1) [9].

**Scheme 1:**
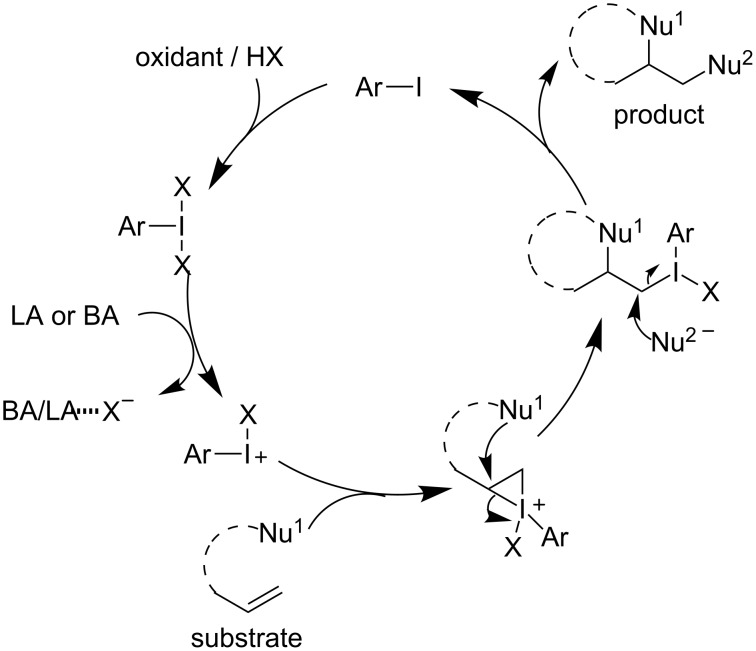
Hypervalent iodine(III)-catalyzed functionalization of alkenes.

The chemistry of hypervalent iodine(III) reagents is now a well-established area in organic chemistry. They are efficient oxidants in many synthetic transformations, such as oxidation of alcohols and phenols, α-functionalization of carbonyl compounds, spirocyclizations, as well as functionalization of alkenes and alkynes [10–17]. In recent years, especially the functionalization of alkenes has attracted much attention [18–20] and in some cases, hypervalent iodine(III) reagents were applied to oxidize transition metals [21–25]. In an alternative way, the electrophilic hypervalent iodine(III) reagents can activate alkenes directly in a metal-free manner. Based on this strategy, dichlorination [26], 1,2-difluorination [27], gem-difluorination [28], aminofluorination [29], dioxygenation [30–31], and diamination [32–33] of alkenes could be achieved. Especially, when a nucleophile-tethered alkene is used, a cyclization product was obtained [34–35], although, stoichiometric amounts of hypervalent iodine(III) reagents were required. Due to the metal-like properties of hypervalent iodine(III), a catalytic variant would be feasible (Scheme 1) [10–17]. In the catalytic cycle, hypervalent iodine(III) can be generated by oxidation of iodoarenes in the presence of a suitable external oxidant.

In 1994, Fuchigami and Fujita reported the first hypervalent iodine(III)-catalyzed reaction, in which the iodine(III) reagent was in situ generated by anodic oxidation under electrochemical conditions [36]. Critical to success of this process is that the oxidation potential of the catalysts should be much lower than those of the substrates and products. In addition, inorganic oxidants and peracetic acids can be used as oxidants as well. In 2005, the Ochiai and Kita groups demonstrated that *m*-chloroperbenzoic acid (*m*CPBA) was a better choice for the in situ generation of hypervalent iodine reagents through oxidation of iodoarenes [37–38]. Based on their studies, various hypervalent iodine(III)-catalyzed reactions were developed [39], including asymmetric variations [40–42]. Due to our interest in the functionalization of alkenes, this review focuses on significant progresses in hypervalent iodine(III)-catalyzed functionalization of alkenes as well as some asymmetric reactions employing a chiral iodoarene.

## Review

### Dioxygenation of alkenes

**Scheme 2:**
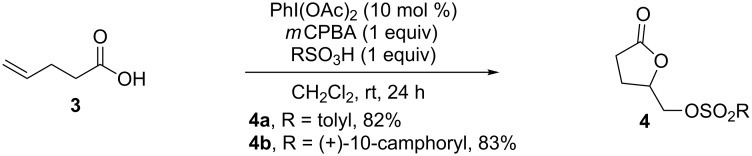
Catalytic sulfonyloxylactonization of alkenoic acids [43].

In 2009, Yan and co-workers reported an efficient catalytic method for the sulfonyloxylactonization of alkenoic acids (Scheme 2) [43], which employed catalytic amounts of hypervalent iodine(III) reagents and *m*CPBA as a stoichiometric terminal oxidant. The cyclization of various alkenoic acids in the presence of sulfonic acids such as *p*-toluenesulfonic acid and (+)-10-camphorsulfonic acid afforded the corresponding sulfonyloxylactones **4** in good yields. A control experiment indicated that the same result was obtained by replacing PhI(OAc)_2_ with PhI. In addition, phosphates were suitable nucleophiles in this reaction, giving phosphoryloxylactones in good yields [44]. A similar catalytic cyclization of unsaturated amides leading to oxazolines and dihydrooxazines was developed, in which Selectfluor was used as a stoichiometric oxidant [45].

**Scheme 3:**
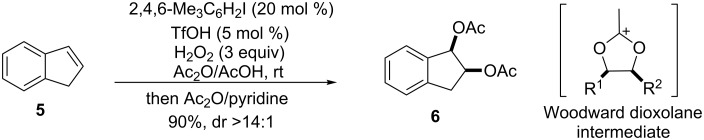
Catalytic diacetoxylation of alkenes [46].

In contrast to the intramolecular cyclization, the intermolecular reaction is much more attractive. Li and co-workers reported a *syn*-diacetoxylation of alkenes, using iodoarenes as catalyst which was oxidized to hypervalent iodine(III) by hydrogen peroxide in the presence of acetic anhydride [46]. As the peroxy compounds can react with alkenes, leading to the *anti*-products via epoxy intermediates, substrates must be added slowly by a syringe pump to provide reasonable yields and diastereoselectivity. The diastereoselectivity of the reaction can be rationalized by Woodward dioxolane intermediates (Scheme 3).

**Scheme 4:**
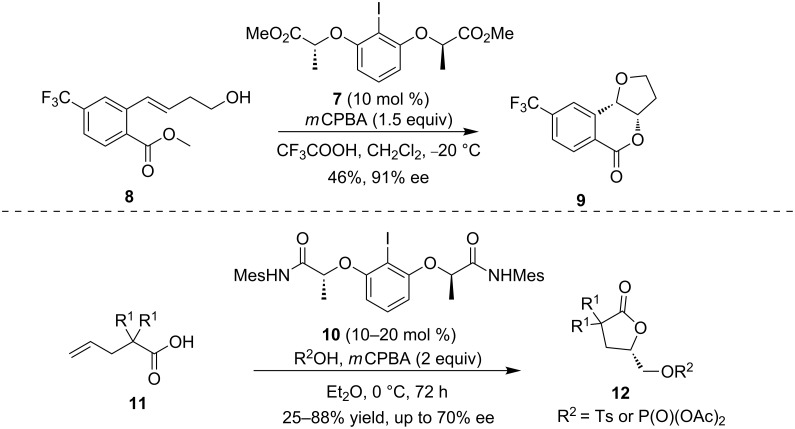
Intramolecular asymmetric dioxygenation of alkenes [48,50].

Stereoselective dioxygenation using catalytic amounts of chiral hypervalent iodine reagents is a comparatively new area in hypervalent iodine chemistry. Fujita and co-workers described a stereoselective oxylactonization reaction in the presence of a chiral hypervalent iodine catalyst [47–48]. Additionally, *m*CPBA and trifluoroacetic acid were utilized as terminal oxidants and activators, respectively. This reaction provided a series of 4-oxyisochroman-1-ones, which are found in natural products and bioactive polyketide metabolites. For example, the reaction of hydroxylated substrate **8** afforded the dihydrofuran-fused isochromanone **9** with up to 91% ee (Scheme 4, top) [48]. The enantioselective control mode is the same as that described in stoichiometric reactions [49]. Recently, Masson and co-workers described an enantioselective iodoarene-catalyzed sulfonyl- and phoshoryloxylactonization of alkenoic acids **11** with additional nucleophiles (Scheme 4, bottom) [50], in which a bisamide chiral precatalyst was applied [51]. This reaction provides an efficient access to various interesting enantioenriched γ-lactones through a tandem sequence in acceptable yields and moderate to excellent enantioselectivities.

**Scheme 5:**
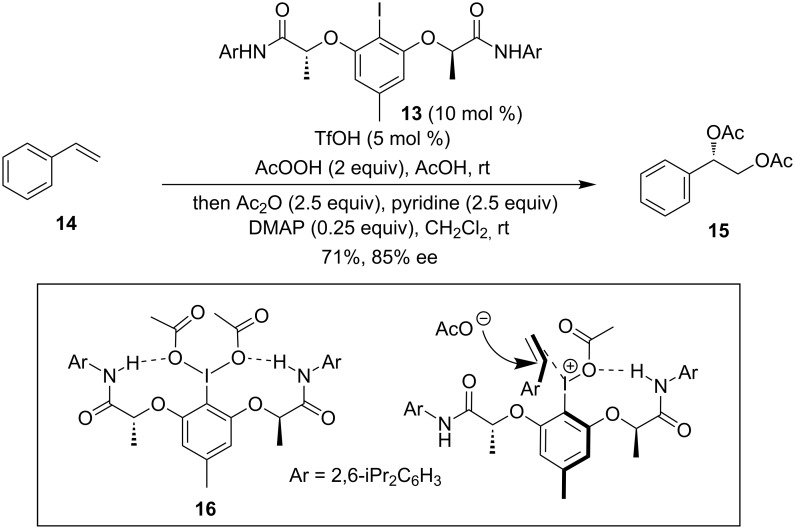
Intermolecular asymmetric diacetoxylation of styrenes [52].

Recently, an intermolecular asymmetric dioxygenation reaction has been disclosed by Muñiz and co-workers (Scheme 5) [52]. They found that the chiral iodoarene catalyst **13**, bearing sterically hindered *N*-arylamido substituents, was essential for the asymmetric induction. The NH group engages hydrogen bonding with the acetoxy groups located at an iodine(III) center to form two nine-membered rings, which were confirmed by the crystal structure of the iodine(III) reagent **16** [53]. The hydrogen bonding effect is crucial to a supramolecular helical chiral environment at the iodine center. Mechanistically, the iodine(III) **16** is activated by triflic acid generating a free coordination site at the iodine(III) center [54]. The coordination of the alkene to the activated iodine(III) center generates the required prochiral face differentiation and the nucleophilic attack of acetates to the exposed *re*-face establishes the *S*-configured benzylic C–O bond.

**Scheme 6:**
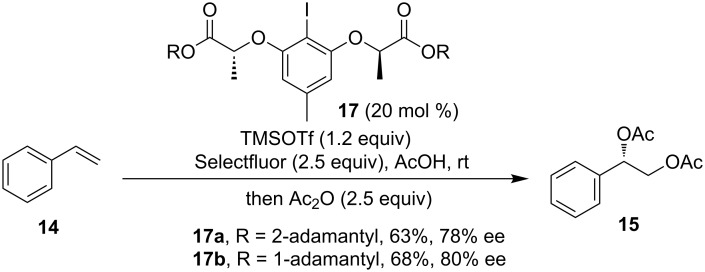
Diacetoxylation of alkenes with ester groups containing catalysts **17** [55].

Without the hydrogen bonding effect, the same reaction with a diester-containing iodoarene catalyst was explored [55]. The sterically hindered adamantyl-substituted catalysts **17** were demonstrated to be efficient to afford the diacetoxylation products in moderate yields and enantioselectivity when using Selectfluor as a terminal oxidant (Scheme 6).

### Diamination of alkenes

**Scheme 7:**
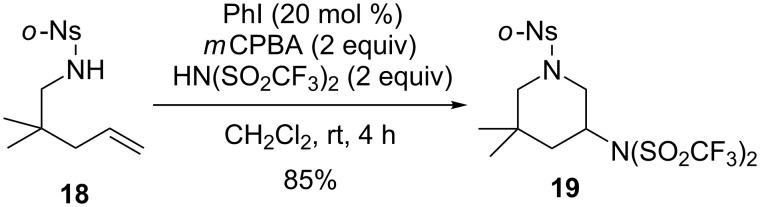
Intramolecular diamination of alkenes [56].

The diamination of alkenes is attractive due to the significance of diamino moieties in diverse fields of the biomedicinal and pharmaceutical sciences. During the study of the hypervalent iodine-mediated intramolecular diamination of alkenes, Blakey and co-workers found that a catalytic version could be achieved in the presence of *m*CPBA to give the same product in 85% yield, which is slightly lower than the yield of the stoichiometric reaction (96%, Scheme 7) [56].

**Scheme 8:**
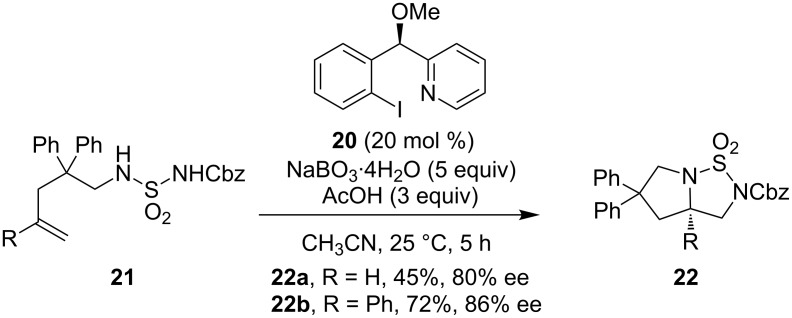
Intramolecular asymmetric diamination of alkenes [57].

Later, Wirth and co-workers developed the first highly stereoselective intramolecular diamination of alkenes using a novel, simple hypervalent iodine(III) catalyst **20** (Scheme 8) [57]. In this reaction sodium perborate was the best terminal oxidant rather than *m*CPBA. Furthermore, the selected protecting group can be removed easily under reducing conditions, providing the free diamine derivatives. However, the substrate scope was limited to alkenes bearing phenyl substituents on the backbone.

**Scheme 9:**
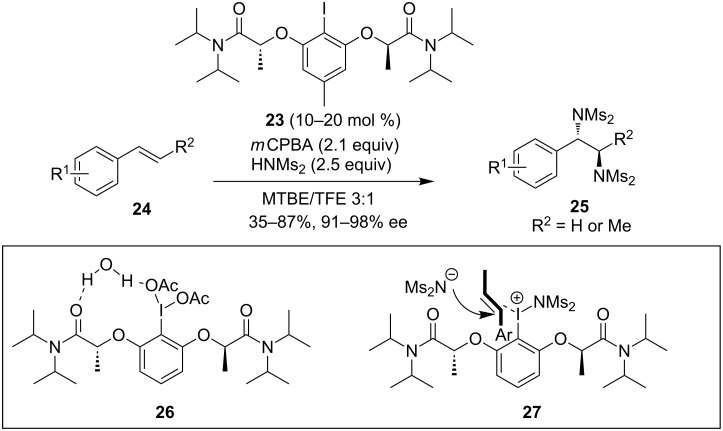
Intermolecular asymmetric diamination of alkenes [58].

On the contrary, the intermolecular diamination of alkenes presented a big challenge under metal-catalyzed conditions, due to the high affinity of the diamine products to metal catalysts. Thus an iodoarene-catalyzed strategy provides a valuable alternative way to the diamination of alkenes. Recently, Muñiz and co-workers reported a chiral iodoarene-catalyzed intermolecular diamination of styrene derivatives (Scheme 9) [58]. An iodoarene precatalyst **23** bearing the tertiary amide on the lactic side chains was the most effective. *m*CPBA was used as a stoichiometric oxidant and bismesylimide as an amine source. It is noteworthy that solvent was a key factor to suppress the undesired epoxidation products. Not only terminal styrenes but also internal alkenes were suitable to this reaction, affording the *anti*-diamination products. The exact mode of stereoinduction with the new catalyst **23** was examined, and the single crystal X-ray structural analysis of **26** revealed that a water molecule engages in double hydrogen bonding to form an 11-membered ring, resulting in the chiral helicity. The helical chirality induced in iodine(III) derivatives of **23** bearing the bislactamide motif was described for **27** with an efficient differentiation of the enantiotopic faces of the styrene substrate. This protocol acted as an asymmetric gateway to the useful vicinal diamines.

### Aminofluorination of alkenes

**Scheme 10:**
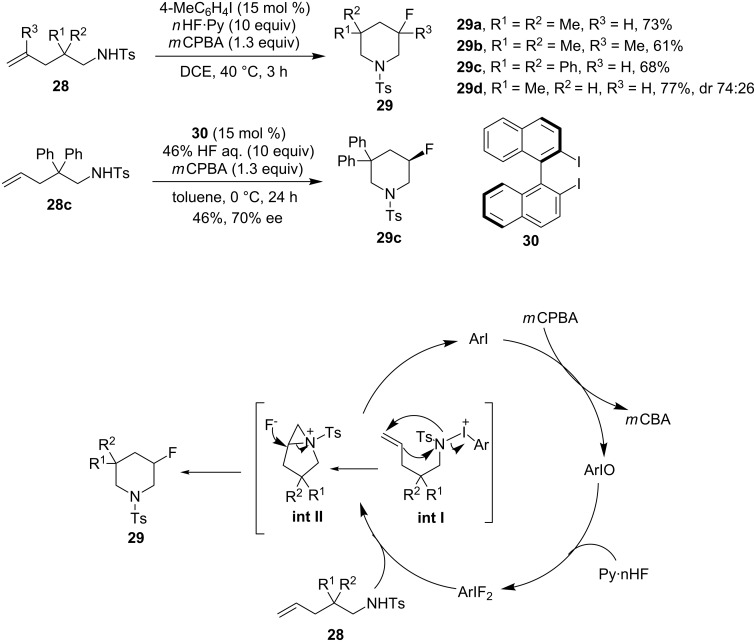
Iodoarene-catalyzed aminofluorination of alkenes [60–61].

(Difluoroiodo)arenes (Ar-IF_2_), which can be prepared from HF with iodosoarenes, are hypervalent iodine compounds suitable for the fluorination of alkenes [59]. Based on Nevado’s stoichiometric aminofluorination [29], Shibata, Kita and co-workers reported the iodoarene-catalyzed aminofluorination of amino-tethered alkenes to yield fluorinated cyclic amines [60]. The mechanism for the catalytic aminofluorination of alkenes is shown in Scheme 10. The iodosyl species, ArI=O, produced by the oxidation of ArI with *m*CPBA, reacted with HF to provide the corresponding in situ-generated difluoroiodoarenes. An aziridinium intermediate **II**, which underwent nucleophilic attack by fluoride on the multisubstituted carbon to afford the *endo*-cyclized products **29**, was proposed in Nevado’s reaction [29]. Employing the binaphthyldiiodide **30** as a catalyst was found to be effective for the asymmetric induction. However, the substrate scope of the reaction was still limited to diphenyl-substituted alkenes. This catalytic system could be applied to the aminofluorination of homoallylamines giving *N*-tosyl-3-fluoropyrrolidines in good to high yields [61].

**Scheme 11:**
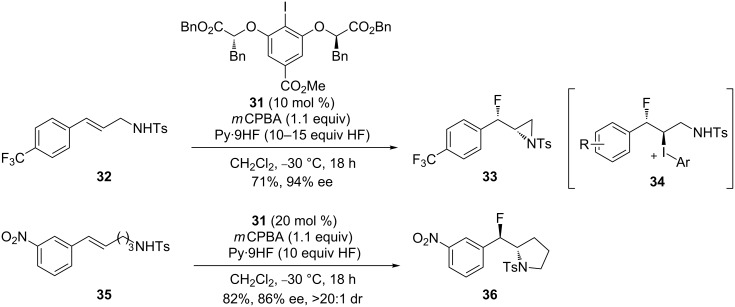
Iodoarene-catalyzed aminofluorination of alkenes [62].

Recently, Jacobsen and co-workers reported the stereoselective synthesis of *syn*-β-fluoroaziridine building blocks via a chiral aryl iodide-catalyzed fluorination of allylic amines (Scheme 11) [62]. On the basis of their previous work, the *C*_2_-symmetric aryl iodide **31** as a catalyst was applied in this reaction. A C(sp^3^)–I(III) intermediate **34**, which was trapped by vicinal nitrogen nucleophiles to form the chiral *syn*-β-fluoroaziridine **33**, was proposed. Both fluoroaziridines **33** and β-fluoropyrrolidines **36** were obtained in good yields and high *anti*-stereoselectivity. However, the reaction of substrates bearing either two or four methylene groups between the alkene and sulfonamide failed to provide the fluoroheterocycles.

### Difluorination of alkenes

**Scheme 12:**
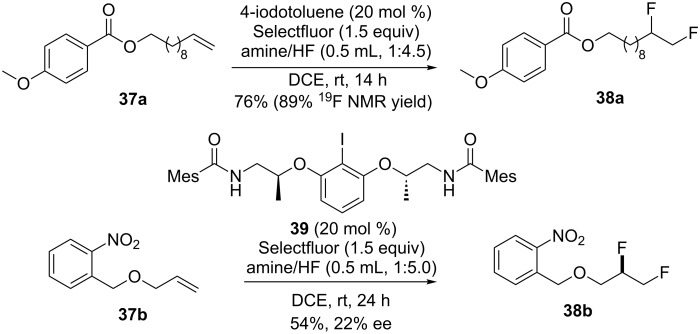
Catalytic difluorination of alkenes with Selectfluor [63].

Hara and co-workers reported a vicinal difluorination of unactivated alkenes in the presence of stoichiometric amounts of difluoro iodotoluene [27]. Based on this seminal work, Gilmour and co-workers reported a catalytic difluorination of alkenes using an inexpensive *p*-iodotoluene as the catalyst and Selectfluor as the terminal oxidant [63]. Terminal olefins proved to be viable substrates for this reaction. It is worth noting that the ratio of amines and HF was important for obtaining reasonable yields. Indeed, excellent ^19^F NMR yields albeit lower isolated yields were obtained in this reaction (Scheme 12). In an attempt to induce enantioselectivity, the chiral aryl iodide derivative **39** only gave a moderate enantioselectivity (22% ee).

**Scheme 13:**
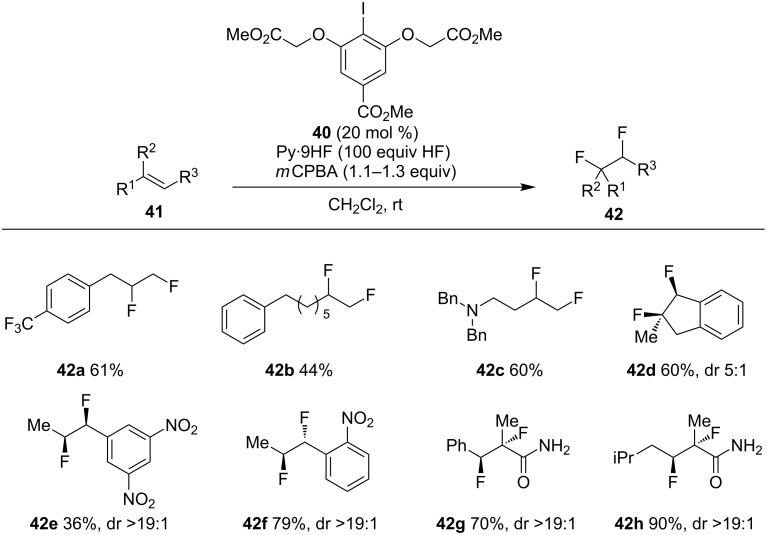
Iodoarene-catalyzed 1,2-difluorination of alkenes [64].

Meantime, a similar work was independently reported by Jacobsen and co-workers, in which the reactive iodoarene difluoride could be in situ generated by oxidation of aryl iodide **40** with *m*CPBA [64]. The reaction showed a wide substrate scope, with toleration of terminal, internal alkenes as well as electron-deficient unsaturated carbonyl compounds (Scheme 13). In general, terminal alkenes were found to undergo 1,2-difluorination **42a**–**c**. The reaction of internal alkenes usually afforded the *syn*-difluorination products **42d** and **42e**. However, the opposite result was observed in the reaction of the *o*-nitrostyrene derivative **42f**, due to the Lewis basicity of the nitro group. These stereochemical outcomes were also observed in the reaction of acrylamides by means of anchimeric assistance.

**Scheme 14:**
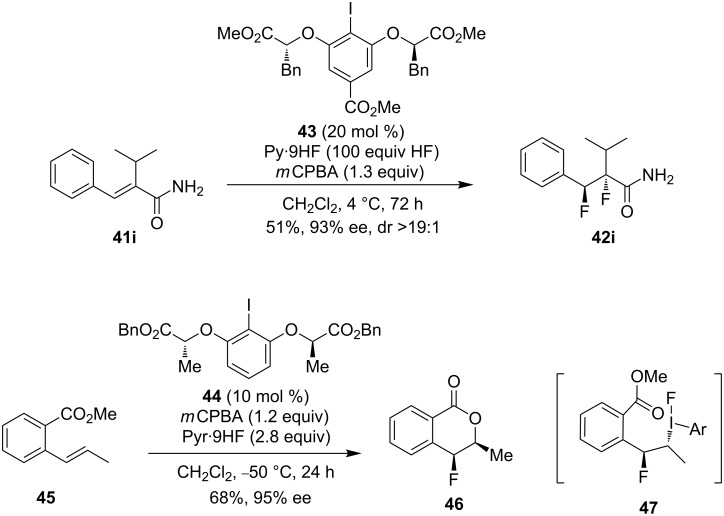
Iodoarene-catalyzed asymmetric fluorination of styrenes [64–65].

Preliminary studies to identify asymmetric variants indicated that, in the presence of lactate-based chiral iodoarene catalyst **43**, the cinnamamide **41i** could be transformed to the corresponding difluorination product **42i** with excellent enantioselectivity and high stereoselectivity, albeit in moderate yields (Scheme 14, top) [64]. Inspired by the propensity for such anchimeric assistance in these reactions, an enantio- and diastereoselective catalytic fluorination was developed by the same group (Scheme 14, bottom) [65] using the lactate-based resorcinol derivative **44** as the catalyst. By this route chiral 4-fluoroisochromanones **46** could be accomplished in high enantio- and diastereoselectivity. The same I(III) intermediate **47** was trapped by an *o*-carboxylic ester group leading to the *syn-*diastereoisomeric outcome.

**Scheme 15:**
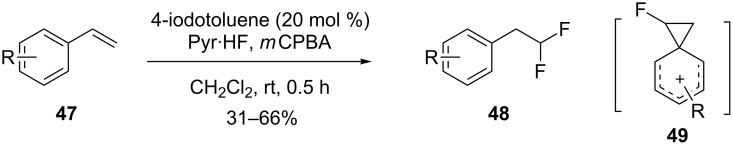
Gem-difluorination of styrenes [67].

An aryl rearrangement might be realized via benzenium ions in the iodine(III)-mediated reactions of styrenes [66]. Oyamada and co-workers reported the synthesis of 2,2-difluoroethylarenes mediated by iodine(III) reagents. Moreover, they found that this fluorination also proceeds with catalytic amounts of the iodoarenes in the presence of *m*CPBA as a terminal oxidant, albeit in lower yields (Scheme 15) [67]. Mechanistically, the 1,2-aryl shift could arise via phenonium intermediates **49** to deliver the geminal difluorination products.

**Scheme 16:**
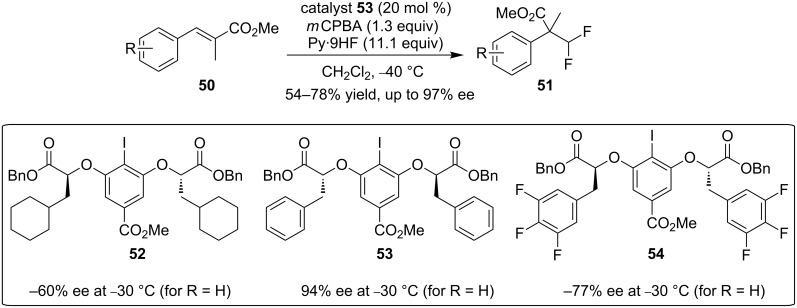
Asymmetric gem-difluorination of cinnamic acid derivatives [68].

Recently, Jacobsen and co-workers reported a highly enantioselective gem-difluorination of various cinnamic acid derivatives through the same oxidative rearrangement (Scheme 16) [68]. During the catalysts screening, they found that the benzylic unit in the catalysts was essential for a high enantioselectivity (**52** vs **53**). Moreover, the more electron-deficient 3,4,5-trifluorophenyl analog **54** was found to be less enantioselective. The authors proposed that the benzylic groups can stabilize the cationic intermediates and/or transition states through cation–π interactions, which play an important role in the stereodifferentiation step.

### Other functionalizations of alkenes

**Scheme 17:**
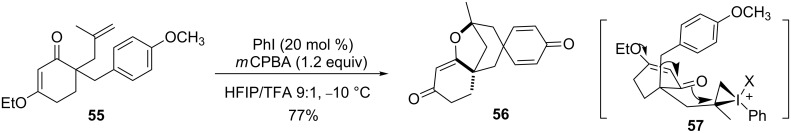
Oxyarylation of alkenes [71].

In addition to heteroatom-containing nucleophiles, electron-rich aromatic groups were also reported as nucleophiles to form the C–C bonds [69–70]. In this context, Lupton, Hutt and co-workers reported an iodobenzene-catalyzed 1,2-olefin functionalization via C–C and C–O bond formation, in which electron-rich aromatic groups and vinylogous esters acting as independent nucleophiles to provide oxabicyclo[3.2.1]octanes (Scheme 17) [71]. Mechanistically, the olefin is activated by iodine(III) to form species **57** which is followed by first a nucleophilic attack from the vinylogous ester, then by the aromatic group, providing the final outcomes.

**Scheme 18:**
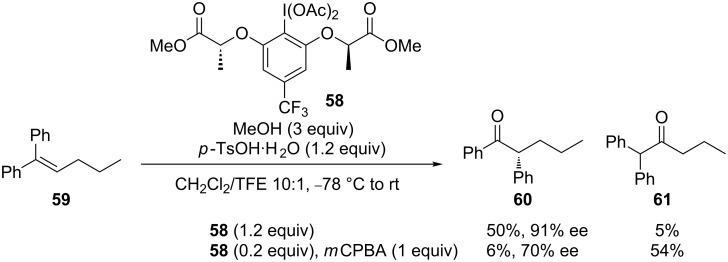
Asymmetric oxidative rearrangements of alkenes [72].

Wirth and co-workers developed an oxidative rearrangement of alkenes to chiral α-aryl ketones, in which electron-deficient chiral lactic acid-based hypervalent iodine reagents were synthesized and applied [72]. The regioselective methoxylation of diphenyl alkene with chiral hypervalent iodine **58** afforded a mixture of **60** and **61** in moderated yield and good enantioselectivity. However, the catalytic reaction afforded the opposite regioselectivity to give rearrangement product **60** in dramatically decreased yield and enantioselectivity (Scheme 18). Similar oxidative rearrangement reactions with haloalkenes generated α-halo ketones [73].

**Scheme 19:**
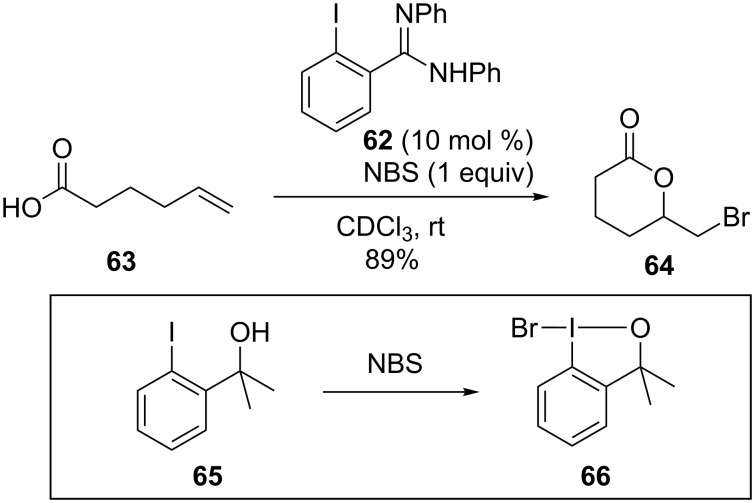
Bromolactonization of alkenes [75].

NBS also oxidizes iodoarene **65** to form the brominating agent **66** [74]. Braddock and co-workers reported an organocatalyzed transformation of electrophilic bromines to alkenes, using *ortho*-substituted iodobenzene **62** as an organocatalyst (Scheme 19) [75]. A control experiment indicated that only trace amounts of products were observed in the absence of iodoarene catalyst (2%). A similar work involving a rearrangement of imides, which delivered α,α-disubstituted-α-hydroxy-carboxylamides, was disclosed by Gulder and co-workers [76].

**Scheme 20:**
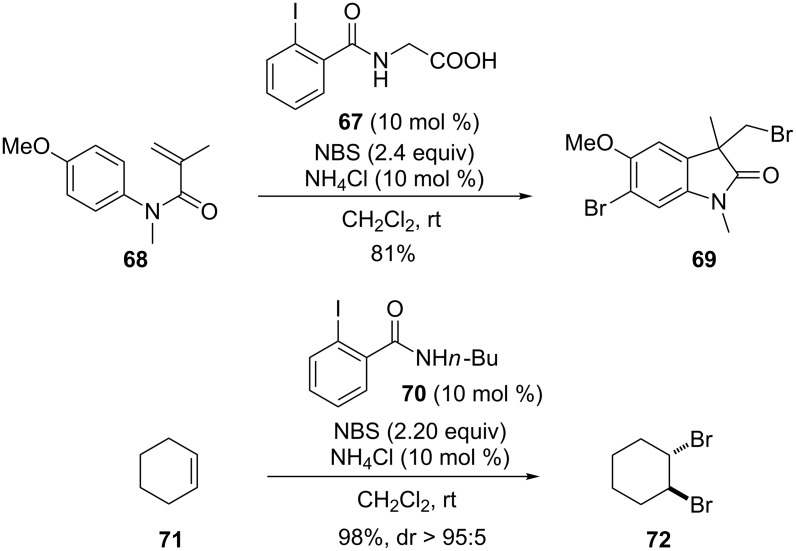
Bromination of alkenes [77–78].

This catalytic system was applied to the bromination of alkenes by Gulder and co-workers. For example, the iodine(III)-catalyzed halocyclization of methacrylamide **68** generated the brominated oxindole **69** (Scheme 20) [77]. In addition, electron-rich aromatics present in the substrates were also brominated. During the screening of the iodoarene pre-catalysts, a dihalogenation product was detected in the presence of iodoarenes bearing electron-donating side chains **70**. A diastereoselective dihalogenation method was established under mild conditions [78]. The authors proposed a radical pathway involving the in situ generation of Br_2_, which opens the avenue for a reliable, ecologically benign, and safe dibromination method.

## Conclusion

In the last two decades, great progress was made in hypervalent iodine(III) catalytic systems. On the basis of these improvements, it is no longer necessary to prepare hypervalent iodine compounds, as the iodide precursors can be used catalytically. The recently developed enantioselective hypervalent iodine(III)-mediated transformations could be a breakthrough for the application of these reagents in chiral synthesis.

As outlined, there have been achieved great advances in the hypervalent iodine-mediated functionalization of alkenes. However, the types of chiral iodoarene catalysts are limited and new chiral iodoarene scaffolds should be developed for highly stereoselective reactions.

**Scheme 21:**
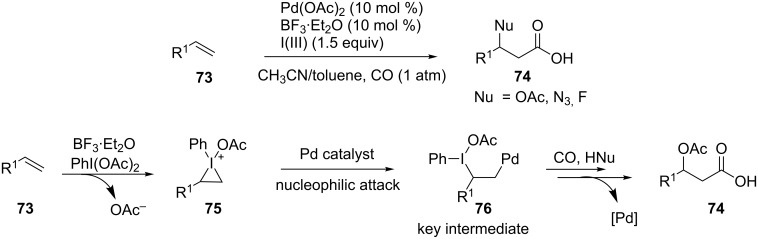
Cooperative strategy for the carbonylation of alkenes [79].

Compared to the diverse reactivity profiles of transition metal-catalyzed functionalization of alkenes, hypervalent iodine (III)-mediated reactions are limited to nucleophilic substitution processes. Recently, Liu and co-workers reported a novel cooperative strategy by combining palladium catalysis and hypervalent iodine-mediated reactions to achieve the intermolecular oxycarbonylation [79], azidocarbonylation [80] and fluorocarbonylation [81] of alkenes. Mechanistic studies showed that PhI(OAc)_2_ is activated by the aid of BF_3_·OEt_2_ and then reacts with an alkene to form a three-membered iodonium ion intermediate **75**. Subsequently, this intermediate is attacked by the palladium catalyst under a CO atmosphere to form the alkyl palladium species **76**. Finally, the reductive elimination at the iodine(III) center and CO insertion into the newly formed C–Pd bond, affords the oxycarbonylation products **74** (Scheme 21). This strategy provides an attractive development tendency in hypervalent iodine(III) chemistry. It is fascinating to realize such transformations with catalytic amounts of iodoarenes as well as chiral iodoarene reagents to induce enantioselectivity.
